# Eating Disorder in a 17-Year-Old Male in a Limited Resource Environment: A Case Report

**DOI:** 10.7759/cureus.103367

**Published:** 2026-02-10

**Authors:** Stewart J Lockett, Jon P Jones, Theodora S Browne

**Affiliations:** 1 Department of Psychiatry, University of Mississippi Medical Center, Jackson, USA; 2 School of Medicine, University of Mississippi Medical Center, Jackson, USA

**Keywords:** adolescent, bulimia nervosa, disordered eating inpatient, eating disorder, family-based therapy, limited resources, male, multidisciplinary care, purging behavior, underserved population

## Abstract

Disordered eating has become a rising concern, as severe cases can result in medical emergencies. Eating disorders in adolescents are often underrecognized, particularly in adolescent males, partly due to gender-related stigma and atypical presentations compared to females. These challenges can be further compounded in regions with limited access to specialized eating disorder treatment services. We present the case of a 17-year-old male brought to our emergency department following a binge-purge episode that was deemed a medical emergency due to bradycardia, orthostasis, low BMI, and electrolyte abnormalities. This case was complicated by diagnostic challenges, including suspected attempts to purge while hospitalized. A multidisciplinary team was assembled to ensure acute stabilization. Family-based therapy was prioritized to assist in medical stabilization. The patient was successfully stabilized and discharged with a structured outpatient plan. This case highlights the diagnostic and treatment challenges of managing adolescent males with eating disorders, especially in states with limited specialized resources. Our findings indicate that collaboration between medical and psychiatric teams can facilitate safe and effective care even in non-specialized settings. Clinicians should maintain a high suspicion for eating disorders in adolescent males and be prepared to treat them in hospital settings without access to specialized resources.

## Introduction

Eating disorders are classified by the Diagnostic and Statistical Manual of Mental Disorders, 5th edition (DSM-5) as involving one or more of the following features: restriction of energy intake relative to requirements, an intense fear of gaining weight or becoming fat, or a disturbance in the way one’s body weight or shape is experienced. This may include an undue influence of body weight or shape on self-evaluation or a persistent lack of recognition of the seriousness of one’s low body weight [1]. These are hallmarked by neurobiological dysregulation of appetite and reward pathways, and psychosocial factors, culminating in maladaptive eating behaviors and impaired energy homeostasis [1]. 

Historically, eating disorders have been more readily recognized in females. Notably, it was not until 2013 that the criterion of amenorrhea (loss of menstrual cycles) was removed from the diagnostic criteria for anorexia nervosa, allowing for more accurate diagnosis in males [2]. Clinically significant disordered eating behaviors that do not meet criteria for anorexia nervosa, bulimia nervosa, or binge eating disorder are classified under “other specified feeding or eating disorders” in the DSM-5. A meta-analysis of 32 studies including 63,181 participants across 16 countries found that 22% of children and adolescents exhibited disordered eating behaviors [3].

According to the National Comorbidity Survey Adolescent Supplement, the lifetime prevalence of eating disorders among U.S. adolescents aged 13-18 years is 2.7%, with males representing approximately 0.1-0.3% for anorexia nervosa, 0.1-0.2% for bulimia nervosa, and 0.3-0.7% for binge eating disorder [4]. A population-based study in Ontario, Canada, reported a 139% increase in eating disorder-related hospitalizations between 2002 and 2019, with the greatest rise observed among males (416%; from 0.2 to 1.1 per 10,000 population), adolescents aged 12-14 years (196%; from 2.2 to 6.6 per 10,000), and those diagnosed with disorders other than anorexia nervosa or bulimia nervosa (255%; from 0.6 to 2.1 per 10,000) [5].

The American Academy of Pediatrics emphasizes that disordered eating is often underrecognized in males and may present differently than in females, with a focus on leanness, weight control, and muscularity. Behaviors such as purging, use of muscle-building supplements, and substance abuse are also common [6]. Adolescent inpatient and residential treatment programs are concentrated in certain metropolitan areas, leaving many regions under-resourced. The purpose of this case report is to highlight the challenges in diagnosing and treating male adolescents with eating disorders, particularly in states with limited healthcare resources.

## Case presentation

The patient is a 17-year-old male with a history of prior suicide attempt, self-injury, and bingeing/purging behaviors, who presented to the emergency department after attempting to run away. He reported consuming two dozen donuts followed by self-induced vomiting, in the context of suicidal ideation with a plan and intent to jump off a bridge. He described a longstanding “weird relationship with food” dating back to childhood, noting early health-conscious behaviors alongside episodes of overeating. While living abroad, he experienced teasing and was called “chubby,” contributing to body dissatisfaction. Around age 16, following a romantic breakup, he developed recurrent cycles of restriction followed by binge eating and purging, interspersed with intermittent periods of remission.

On examination, he appeared markedly underweight, with bilateral parotid gland enlargement, and healed linear scars on both wrists consistent with prior self-injury. Vital signs demonstrated hypotension (BP 93/60 mmHg), profound bradycardia (HR 43 bpm), and mild hypothermia (97.5°F/36.4°C). Growth parameters revealed severe underweight status: height 1.85 m (6′0.84″), weight 55.2 kg (121 lb 11.1 oz), BMI 16.1 kg/m². Laboratory report before treatment revealed mild metabolic abnormalities consistent with malnutrition, meeting criteria for medical instability and necessitating inpatient admission for stabilization and monitoring.

He was admitted with a provisional diagnosis of unspecified feeding or eating disorder, with consideration for anorexia nervosa, binge-eating/purging type. Due to limited local specialized resources, he was managed on the pediatric medicine service with psychiatric consultation. A multidisciplinary team, including pediatrics, psychiatry, nutrition, psychology, and social work, coordinated care.

Management and outcome

The patient was formally diagnosed with anorexia nervosa, restricting/purging type. He was initiated on a pediatric eating disorder protocol, including strict intake/output monitoring, daily weight checks, bed rest, and 1:1 observation to reduce the risk of purging. Nutrition implemented a graded high-calorie meal plan with Boost supplementation three times daily. Electrolytes and renal function were monitored every 12 hours for refeeding syndrome, which did not occur. Symptom management included MiraLAX®, Pericolace®, Pepcid®, Tylenol®, and ondansetron for constipation, nausea, vomiting, headaches, and abdominal pain.

During hospitalization, he exhibited emotional fluctuations, including meal refusal, distress, and attempts to leave the unit. Family-based therapy emphasized structured supervision and routines. He also experienced diarrhea and vomiting from a rhinovirus/enterovirus infection, treated symptomatically. Despite these challenges, he gradually tolerated meals, gained minimal weight, and his vital signs stabilized.

After 10 days, he reached clinical stability, including stable lab work, along with reaching his caloric intake with no binging/purging episodes noted. His family was offered continued inpatient treatment, but they declined. A multidisciplinary discharge meeting with the patient and his family established home care instructions, including supervised meals, avoidance of exercise, social media, and purging behaviors, as well as continuation of boost supplementation to achieve caloric goals. He was discharged on hospital day 11 with a BMI of 16.5 kg/m², with a goal BMI of 18.0 kg/m². Outpatient follow-up included weight checks three times per week and psychology sessions twice weekly, later transitioning to a community counseling program. Post-discharge, he demonstrated stable weight trends, improved vitals, and gradual psychosocial adjustment per pediatrician notes.

## Discussion

This case underscores the diagnostic challenges and resource limitations in treating male adolescents with eating disorders. The patient’s social pressures and internalized ideals of thinness reflect risk factors often underrecognized in males. Male eating disorders are frequently undiagnosed due to atypical presentations, including a focus on muscularity rather than weight, less obvious weight loss, and differing patterns of body dissatisfaction. The term muscle dysmorphia (“bigorexia”) is used to describe males who perceive their bodies as too small, weak, or insufficiently muscular [7]. In this case, the patient frequently described being praised for his athleticism and physique, further complicating the psychological context of his eating disorder.

The patient’s initial resistance to treatment before medical improvement highlights the nonlinear course of recovery in adolescent eating disorders. The absence of local specialized eating disorder programs in resource-limited areas further complicates timely diagnosis and intervention. In community samples, only 19-36% of individuals with eating disorders ever sought or received specialist treatment, with a median delay of 10-15 years from symptom onset to help-seeking [8]. 

Early recognition through careful screening, family engagement, and innovative approaches such as telepsychiatry can mitigate these barriers [9]. Screening in primary care should consider the complex relationship between food, physical activity, and body image in adolescent males, including sports and resistance training.

**Figure 1:**
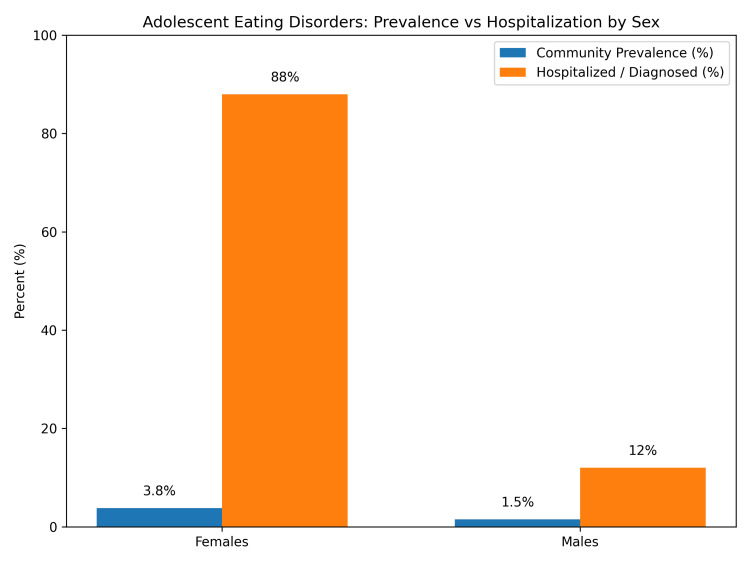
Adolescent eating disorders: prevalence vs. hospitalization by sex The image displays a comparison of the community prevalence and proportion of diagnosed/hospitalized eating disorders in adolescents by sex. Community prevalence (blue bar) shows community prevalence in comparison to the diagnosed/hospitalized proportion (orange bar) by sex. The figure highlights the discrepancy between community prevalence and clinical recognition among males. The figure was created by the authors using data derived from previously published sources [10,11].

As illustrated in Figure 1, although females have roughly twice the prevalence of disordered eating compared with males, they are hospitalized or formally diagnosed at nearly seven times the rate [10,11]. This highlights the difficulties families, clinicians, and society face in recognizing and diagnosing eating disorders in males. In this case, another difficulty was the family choosing not to stay inpatient for further treatment, which would have given access to continued multidisciplinary care. 

The Eating Disorder Examination Questionnaire (EDE-Q) was originally developed for females. While useful for screening males, it may not fully capture male-relevant domains of disordered eating, and measurement bias is possible. In a clinical sample of 245 male patients and 205 male controls, receiver operating characteristic (ROC) curve analysis identified an optimal clinical cutoff score of 1.68 for males, with sensitivity and specificity both at 0.77, indicating moderate-to-good validity [12]. These findings suggest that while the EDE-Q can be helpful in males, its original cutoffs were derived from female samples, raising questions about validity in male populations. Male-specific assessments, such as the Eating Disorder Assessment for Men (EDAM), capture constructs like drive for muscularity, which standard tools may miss, though research comparing these tools with the EDE-Q is limited [13].

Family-Based Treatment (FBT) is recommended as first-line therapy for adolescents with eating disorders by the American Psychiatric Association and the American Academy of Pediatrics [6]. In a randomized controlled trial comparing FBT to Adolescent-Focused Individual Therapy (AFT) in adolescents with anorexia nervosa, short-term remission rates were similar. However, FBT demonstrated significantly higher full remission rates at 6- and 12-month follow-up [14,15]. Systematic reviews and meta-analyses have confirmed these findings [15]. When in-person FBT is unavailable, virtual FBT, cognitive-behavioral therapy, and systemic family therapy have shown efficacy in promoting weight restoration, improving eating behaviors, and addressing psychological factors, even in resource-constrained environments [6].

## Conclusions

This case highlights the diagnostic challenges and delays in care commonly experienced by male adolescents with eating disorders, which often follow an insidious course and present atypically. Traditional screening tools may inadequately capture male-specific symptomatology, including preoccupation with size, muscularity, and exercise-driven purging behaviors, contributing to underrecognition and delayed diagnosis. This case further illustrates the difficulty in accessing appropriate care, as the patient’s illness progressed to a life-threatening stage prior to treatment, reflecting broader systemic limitations, including the scarcity of specialized inpatient eating disorder programs in many regions of the United States. In resource-limited settings, early identification through collaboration among families, primary care providers, and multidisciplinary teams, including psychiatry, psychology, pediatrics, nutrition, and social work, is essential. Following medical stabilization, timely initiation of evidence-based interventions such as Family-Based Therapy, delivered in person when possible or supplemented by virtual modalities, may support sustained recovery. Strengthening screening practices, family education, multidisciplinary collaboration, and innovative use of resources such as telepsychiatry has the potential to improve medical and psychiatric outcomes and reduce morbidity and mortality in this underserved population.
